# Three-Class Differential Diagnosis among Alzheimer Disease, Frontotemporal Dementia, and Controls

**DOI:** 10.3389/fneur.2014.00071

**Published:** 2014-05-12

**Authors:** Pradeep Reddy Raamana, Howard Rosen, Bruce Miller, Michael W. Weiner, Lei Wang, Mirza Faisal Beg

**Affiliations:** ^1^School of Engineering Science, Simon Fraser University, Burnaby, BC, Canada; ^2^Memory and Aging Center at University of California, San Francisco, CA, USA; ^3^Department of Radiology, VA Medical Center at University of California, San Francisco, CA, USA; ^4^Feinberg School of Medicine, Northwestern University, Chicago, IL, USA

**Keywords:** differential diagnosis, Alzheimer, frontotemporal disease, multi-class, ventricle

## Abstract

Biomarkers derived from brain magnetic resonance (MR) imaging have promise in being able to assist in the clinical diagnosis of brain pathologies. These have been used in many studies in which the goal has been to distinguish between pathologies such as Alzheimer’s disease and healthy aging. However, other dementias, in particular, frontotemporal dementia, also present overlapping pathological brain morphometry patterns. Hence, a classifier that can discriminate morphometric features from a brain MRI from the three classes of normal aging, Alzheimer’s disease (AD), and frontotemporal dementia (FTD) would offer considerable utility in aiding in correct group identification. Compared to the conventional use of multiple pair-wise binary classifiers that learn to discriminate between two classes at each stage, we propose a single three-way classification system that can discriminate between three classes at the same time. We present a novel classifier that is able to perform a three-class discrimination test for discriminating among AD, FTD, and normal controls (NC) using volumes, shape invariants, and local displacements (three features) of hippocampi and lateral ventricles (two structures times two hemispheres individually) obtained from brain MR images. In order to quantify its utility in correct discrimination, we optimize the three-class classifier on a training set and evaluate its performance using a separate test set. This is a novel, first-of-its-kind comparative study of multiple individual biomarkers in a three-class setting. Our results demonstrate that local atrophy features in lateral ventricles offer the potential to be a biomarker in discriminating among AD, FTD, and NC in a three-class setting for individual patient classification.

## Introduction

Frontotemporal dementia (FTD), frontal variant of frontotemporal lobar degeneration (FTLD) and Alzheimer’s disease (AD) are two common forms of dementia with distinctive etiologies but share clinical symptoms and the cognitive impairments (1–6). Many patients with pathologically confirmed FTD had been clinically diagnosed with AD during life (7) and 10–40% of patients clinically diagnosed with FTD are found to have AD postmortem (8). The NINCDS-ADRDA criteria for diagnosing probable AD have a sensitivity of 93% but a specificity of only 23% in distinguishing it from FTD as most patients with FTD also fulfilled NINCDS-ADRDA criteria for AD (9). Hence, an accurate discrimination between AD and FTD has important implications for prognosis and symptomatic treatment (10–13).

Much research on discriminating between AD and FTD utilized morphometric features derived from whole-brain magnetic resonance (MR) images. Voxel-based morphometry (VBM) was used to compare patterns of gray matter loss (14–16) and these studies have identified various discriminatory regions between AD and FTD. Patterns of cortical thinning (17) also have been found to be discriminatory between AD and FTD. The study presented in Ref. (14) made use of features obtained from VBM from the whole-brain and constructs high-dimensional features from the discriminating patterns of GM and white matter (WM) volumes distribution. The whole-brain atrophy rate was also found to be similar in FTD and AD (18).

Apart from whole-brain MRI-based features, atrophy and shape deformity of individual structures have also been studied. For example, cross-sectional volume of hippocampus is found to have large overlap in the FTLD and AD groups (19, 20) whereas longitudinal rates of atrophy are found to be discriminatory (19). Asymmetry in amygdaloid atrophy is also found to be discriminatory between FTLD and AD relative to the control group (20, 21). Shape features of neostriatum structures’ caudate nucleus and putamen have also been studied in Ref. (22) and based on pair-wise comparisons, this study reported significant differences in shape of caudate nucleus and putamen in FTD and AD compared to controls; however the utility of these measures in a classification system was not reported.

Besides structural MRI, features have also been derived from other imaging modalities, e.g., regional patterns of WM degradation (relative to controls) have been studied in discriminating FTD from AD (23) and the authors of this study found that WM degradation seems to be more prominent in FTD than in AD. Also features obtained from positron emission tomography with [^18^F] fluorodeoxyglucose (FDG-PET) and Pittsburgh compound B (PiB-PET) (24–29) provide useful features on metabolic impairment and amyloid deposition regionally to allow for discrimination. Longitudinal biomarkers such as the rate of lobar atrophy (30) and atrophy rates in hippocampus and cingulate gyrus (19) were found to be discriminatory between FTD and AD. There were also studies combining features from multiple modalities (31) that identified characteristic patterns for AD and FTD. Although results from functional imaging modalities and longitudinal studies have been encouraging, they require availability of specialized scanners, or multiple repeat visits, which may be cost prohibitive or not readily available. It is therefore of interest to investigate if structural MRI at one time-point can provide morphometric features that can be used for discrimination.

There are also a few limitations associated with the previous studies available in literature. Most studies so far combined the three variants of FTLD into one umbrella diagnostic group to be compared with AD (14, 22, 32), whereas some studies focused only on SD (16, 33, 34) and some on PNFA alone (35, 36). However, there have been only few studies on behavioral variants FTLD (bvFTD) alone. It is important to note that almost every study presented results from pair-wise classification experiments only, e.g., experiments compared only AD vs. normal controls (NC), FTD vs. NC, or a direct AD vs. FTD (14, 17, 30, 37) and sometimes classification results were not presented (22). When a classifier was trained on only two classes, e.g., AD and FTD, the classifier cannot decide if the patient being tested belongs to an entirely different third class, for example, the normal control group, or, at the very least, introduces a bias into its prediction (38, 39). This can lead to a normal control brain MR image getting misclassified as AD or FTD when a classifier trained only on two classes AD and FTD is used. The novel idea proposed here is to discriminate among the three classes simultaneously.

Moreover, the predictive value reported in the previous studies for various features were based on cross-validation (CV) experiments. The performance estimates from CV can result in biased estimates (40), more so when the parameters are optimized using CV (41) and in the presence of small sample sizes (42). Hence, it is important to estimate the performance on an unseen separate test set, which in addition enables comparison of different biomarkers’ performance on the same test set.

In this work, we present a direct three-class study in discriminating among AD, FTD, and NC using biomarkers extracted from structural MRI data alone. The different biomarkers we study are volumes, intrinsic shape features, and extrinsic shape features of the hippocampus and the lateral ventricle. Intrinsic shape features are those extracted for each structure independent of other subjects using a fixed method. Extrinsic shape features are computed with respect to an external reference, e.g., an atlas or template. These biomarkers are studied in a three-class setting directly, using the same classifier to enable comparison of their diagnostic value. Unlike previously published studies that present CV results, the predictive value in our study is assessed on a separate test set. To our knowledge, such an intrinsic three-class study using an independent test set to assess the predictive value has not been published.

## Materials and Methods

Thirty-four patients diagnosed with AD, 30 patients diagnosed with behavioral variant FTLD (bvFTD or simply referred to as FTD in this paper, being the most common variant), and 14 age-matched NC subjects were included in the study (see Table 1). When selecting the healthy controls, the only criterion imposed was to age-match them to that of AD and bvFTD subjects, as the AD and FTD subjects in this dataset are relatively younger (mean age about 56 years, compared to the literature where the mean age of AD/FTD is around 75 and 65 years, respectively). The patients with FTD and AD were recruited from the Memory and Aging Center of the University of California, San Francisco as previously described (17). All patients were diagnosed based upon information obtained from an extensive clinical history and physical examination. FTD was diagnosed according to the consensus criteria established by Neary et al. (1). Patients with FTD who had motor neuron disease-related symptoms were excluded. Patients with AD were diagnosed according to the NINCDS/ADRDA criteria (43). All subjects received a standard battery of neuropsychological tests, including assessment of global cognitive impairment using the mini-mental state examination (MMSE) (44) scores and global functional impairment using the clinical dementia rating (CDR) scale (45). A modified version of the trail-making test (TMT) was used to assess executive functions (46). MRI data were visually inspected by a radiologist to rule out major neuropathologies other than neurodegeneration, such as tumor, stroke, and severe WM disease. All subjects, or their guardians, gave written informed consent before participating in the study, which was approved by the Committees of Human Research at the University of California and the VA Medical Center at San Francisco.

**Table 1:** **Demographics of the cohort**.

Whole dataset	*N*	Age (years) mean ± SD	Gender M + F
bvFTD	30	57.81 ± 3.36	15 + 15
Probable AD	34	55.45 ± 3.06	12 + 22
Normal controls	14	55.40 ± 4.72	5 + 9

### Data acquisition

MRI data were obtained on a 1.5-T Siemens Vision™ System (Siemens Inc., Iselin, NJ, USA), using a standard quadrature head coil. Structural MRI data were acquired using a double spin echo (DSE) sequence and a volumetric magnetization-prepared rapid gradient echo (MPRAGE) T1-weighted sequence. The parameters of MPRAGE T1-weighted images were: TR/TE/TI = 10/7/300 ms, 15° flip angle, 1.00 mm× 1.00 mm in-plane resolution, and 1.40 mm thick coronal partitions and oriented orthogonal to the image planes of DSE.

### Image processing

The subcortical segmentations of hippocampus and lateral ventricles for each target are obtained using multi-atlas fusion (47–49). We chose two atlases each from the small, medium, and large ventricular sizes in the atlas library. Then each subject is segmented using those six atlases via registration-based segmentation approach called FreeSurfer-initiated large deformation diffeomorphic metric mapping (FS + LDDMM) (50, 51). In the automated FS + LDDMM pipeline, FreeSurfer (FS) [(52), using version 4.5.0] first provides rough, initial segmentation of 37 brain regions including the hippocampus and the ventricles in each hemisphere. The FS segmentation of the target is then registered to the atlas image using an intensity-based affine registration to align the local volume of interest for subsequent non-rigid LDDMM registration (51). A bounding box, predefined in the atlas space using the extents of the atlas FS labels plus 12 voxel padding, is then used to generate a sub-volumes region-of-interest (ROI). Intensity normalization of the atlas and target ROIs is then used to match the global median cerebrospinal fluid (CSF) and WM intensities. Finally, the roughly aligned ROI is registered to the atlas image using the LDDMM registration to obtain the segmentation. The resulting segmentations from different atlases are fused to obtain the final segmentation (49). The segmentation for each subject is visually inspected for any inaccuracies. The minor segmentation errors (slight under/overestimation of boundary in few slices) were corrected and the marginally inaccurate segmentations were excluded from the study.

### Feature extraction

In this study, the following three features are extracted from the segmentations of the hippocampus and lateral ventricles:

#### Volumes

This feature is to capture the gross atrophy in the particular structure. This is a global measure in the sense that it is single quantity for the whole shape.

#### Laplacian invariants

This feature is to capture changes in the *intrinsic* shape of the structure. This feature is also a *global* feature in the sense it does not contain any spatial information.

#### Surface displacements

This feature is to capture changes in the *extrinsic* shape of the structure. These features are computed with respect to a fixed atlas to capture local changes on the boundary of the structure and can also be visualized. These features are *local* in the sense that they contain spatial information of the boundary, which is a very useful property as will be seen later.

Both the volumes and the Laplacian invariants are computed using the binary volumetric segmentations obtained from the multi-atlas fusion and the surface displacements were computed from the corresponding surfaces obtained from the injection (see Surface Displacements via Template Injection). The extraction of these features is described in detail in the sections below.

## Volumes

**Figure 1:**
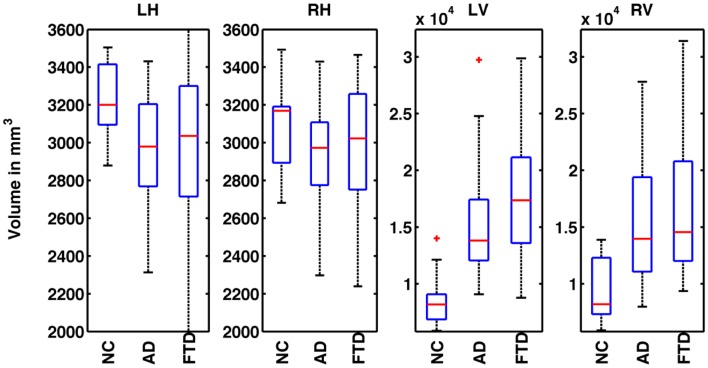
**Distribution of normalized volumes of hippocampi and lateral ventricles for AD, FTD, and NC groups**. The title of the plot identifies the structure for which the volumes are plotted (e.g., LH for left hippocampus, RH for right hippocampus, LV for left ventricle, and RV for right ventricle). In plots 1 and 2, the distributions of volumes for FTD and AD are slightly below the NC group reflecting overall hippocampal atrophy. However, the distributions of FTD and AD volumes overlap and hence, FTD and AD individuals cannot be discriminated based on hippocampal volumes alone. In the plots 3 and 4, the distributions of ventricular volumes for FTD and AD are slightly above that of NC reflecting ventricular expansion. The distributions of FTD and AD volumes overlap, hence FTD and AD individuals cannot be discriminated based on ventricular volumes alone.

The volume of each structure is computed from its binary segmentation obtained from the multi-atlas fusion. These volumes are normalized (53) with respect to the intracranial volume (ICV) obtained from the FreeSurfer (52) parcelation:

$$V_{\text{norm}}^{i}=V_{\text{abs}}^{i}-k\left(\text{IC}{\text{V}}_{i}-\text{IC}{\text{V}}_{\text{mean}}\right)$$

where *V^i^* is the absolute volume of structure and ICV*^i^* is ICV for subject *i*; *k* is the regression coefficient between each subject’s structural volume and its ICV and ICV_mean_ is the mean ICV computed from the NC used in this study. The distribution of normalized volumes of hippocampi and ventricles are shown in Figure 1.

## Laplacian Invariants

Given a bounded domain Ω of general structure in *R*^3^, the eigenfunctions/eigenvalues of the domain found from solving the Helmholtz equation Δ*u* + λ*u* = 0 in Ω with Dirichlet boundary condition *u* = 0 on its boundary γ give the Fourier-like modes based only on the geometry of the domain. The direct computation of the eigensystem of the Laplace operator *L* is difficult since *L* is unbounded. This difficulty is avoided by finding an integral operator commuting with the Laplacian, without imposing a strict boundary condition *a priori* (54). Although the sequence of eigenvalues 0 < λ_1_ < λ_2_ ≤ … ≤ λ_*k*_ ≤ … → ∞ of the Laplacian *L* on the domain Ω is not enough to completely and exactly specify the structure of the domain Ω (55), the following key properties of these Laplacian eigenvalues are shown to provide a very useful set of features to discriminate and cluster structures (56–59):

(1) The eigenvalues are preserved if the underlying domain Ω is translated or rotated (60, 61).
(2) Based on a property of *domain monotonicity*, the ratio of two eigenvalues is found to be invariant with respect to scaling of the underlying domain (56).

Accordingly, for a given binary image Ω, three sets of pose and scale invariant features have been suggested (56):

$$\begin{array}{llll} & F_{1}\left(\Omega \right)\equiv \left(\frac{\lambda_{\text{1}}}{\lambda_{\text{2}}}\text{,}\frac{\lambda_{\text{1}}}{\lambda_{\text{3}}}\frac{\lambda_{\text{1}}}{\lambda_{\text{4}}},\ldots ,\frac{\lambda_{1}}{\lambda_{n}}\right),\; & & \;\\ & F_{2}\left(\Omega \right)\equiv \left(\frac{\lambda_{\text{1}}}{\lambda_{\text{2}}}\text{,}\frac{\lambda_{\text{2}}}{\lambda_{\text{3}}}\frac{\lambda_{\text{3}}}{\lambda_{\text{4}}}\text{,}\ldots ,\frac{\lambda_{n-1}}{\lambda_{n}}\right),\;\text{and}\; & & \;\\ & F_{3}\left(\Omega \right)\equiv \left(\frac{\lambda_{\text{1}}}{\lambda_{\text{2}}}-\frac{d_{1}}{d_{2}},\frac{\lambda_{\text{1}}}{\lambda_{\text{3}}}\text{-}\frac{d_{1}}{d_{3}},\frac{\lambda_{\text{1}}}{\lambda_{\text{4}}}-\frac{d_{1}}{d_{4}},\ldots ,\frac{\lambda_{\text{1}}}{\lambda_{\text{n}}}-\frac{d_{1}}{d_{n}}\right)\; & & \; \end{array}$$

where *n* is the number of features we wish to use for our recognition scheme, and *d*_1_ < *d*_2_ ≤ *d*_3_ ≤ … ≤ *d_n_* are the first *n* eigenvalues (counting multiplicity) of a sphere. The values of *F*_1_(Ω) and *F*_2_(Ω) are in the unit cube, and those of *F*_3_(Ω) are in the interval [−1, 1]. The descriptor *F*_3_ can be interpreted as a measure of the deviation of the structure Ω from a sphere. These sets of features have been shown to be tolerant of boundary noise and deformation and to have good inter-class discrimination capabilities (56, 62). The optimal number *n* of computed features depends on the problem being addressed and is determined experimentally. In this study, we derived the structure invariant features using this formula: λ*_i_*_−1_/λ*_i_, i* = 2: *n* using the first *n* = 100 eigenvalues. We chosen *n* = 100 for Type 2 based on its robust performance in our previous experiments (62).

## Surface Displacements via Template Injection

**Figure 2:**
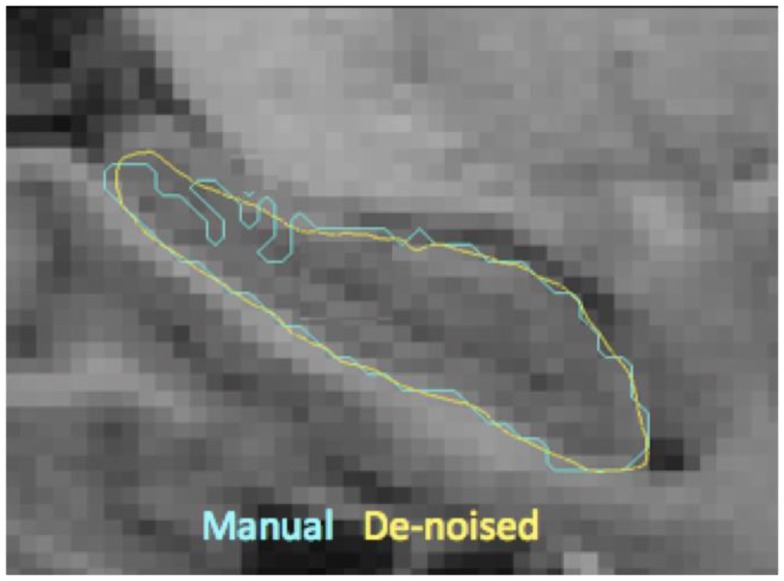
**Shown here are the boundaries of manual delineations (in cyan) of hippocampus and the corresponding injected surfaces (in yellow) overlaid on a sagittal MR slice (zoomed in)**. This visualization illustrates the resulting boundary smoothness after template injection, which is a desirable property prior to extraction of shape features.

In the previous sections, we have extracted intrinsic features that quantify the global volume and Laplacian invariants of the subcortical structures. Here, we extract features that quantify the amount of deformation for an atlas when registered to an individual target. In order to precisely measure the deformation caused by the disease, we present here a *template injection* approach. For each target image *S_i_* in the cohort:

1. We rigidly register the subcortical segmentation *H_i_* to that of the template *T^W^* to obtain a rigid transformation *A_i_*.
2. Transform all the segmentations *H_i_* using this rigid transformation *A_i_* to bring them into a rough alignment with the template.
3. For each target structure *H_i_*, we register template binary segmentation and target binary segmentation to obtain the LDDMM mapping φ*_i_* (51). We inject the template surface *H^A^* of that structure on to the target binary segmentation using φ*_i_* which establishes vertex correspondence between the two surfaces of the target and the template. Note that the correspondence established is not anatomic, but based on the textural information in the vicinity, which most often is accurate. Owing to the diffeomorphic properties of φ*_i_*, the resulting surface will be smooth as shown in Figure 2.
4. At each template vertex *v_j_* of a target *S_i_*, we compute the displacement *d_i_* using *d_i_*(*v_j_*) = φ(*v_j_*) − *v_j_*. Here, *d_i_* is the Euclidian distance between the original and the displaced vertices, capturing displacements in all directions, into a single scalar value with units in millimeter.

The set of these displacements forms a feature vector. A close variant of template injection approach has been presented for the purposes of generation, denoising, and construction of momentum maps (63), whereas our approach uses injection to establish vertex correspondence thereby providing surface displacement features.

### Dimensionality reduction

**Figure 3:**
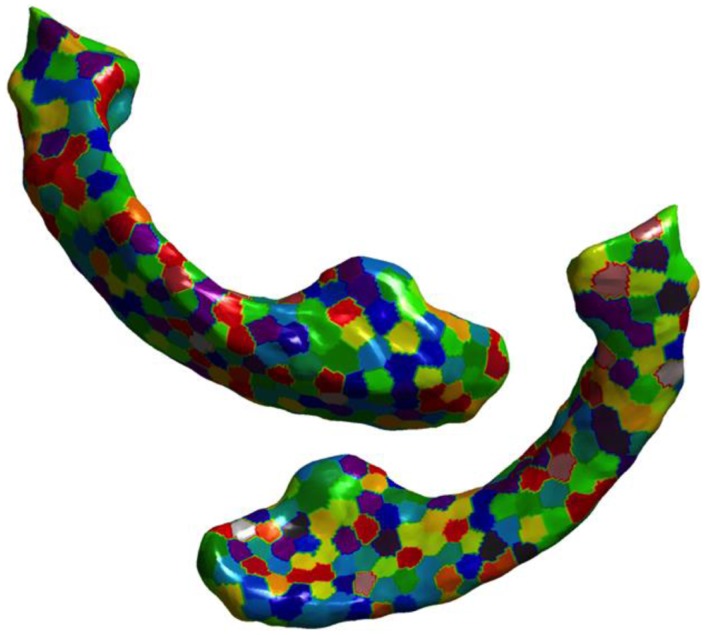
**Visualization of partitions is shown on the ventricular surface of the atlas**. The surface is partitioned into 300 partitions via *k*-means clustering of vertices. In lateral ventricles as well, we can see that the partitions are small enough to capture the variation in the deformation in the curved areas.

The number of vertices on the atlas surface is usually large (e.g., 6000+ for hippocampus and over 30,000 for lateral ventricles). This leads to a curse of dimensionality when performing classification. But we also know that the features are spatially smooth. With the assumption of spatial smoothness, we propose to partition the surface of the subcortical structure into a small number of partitions by clustering vertices with *k*-means clustering of vertex coordinates. The average of the displacements for various vertices in each of these partitions represents the displacement for each partition. This simple approach not only reduces the dimensionality of the features but also does it in an *anatomically meaningful* way. Other dimensionality reduction methods such as PCA transform the features to a different abstract space making interpretation of their output difficult in anatomically relevant terms. Based on preliminary experiments evaluating the resolution of this partitioning (*N* = number of patches on the surface) in its ability to capture local changes, we choose to partition the hippocampal and ventricular surfaces using *N* = 300 partitions, which seems to capture local changes in atrophy with uniformly sized patches, without producing too many small patches. The uniformity in size of the patches is a natural result of *k*-means process, and was not enforced. The visualization of such a partition of hippocampus and ventricles into *N* = 300 partitions is shown in Figure 3 and the mean displacements in different diagnostic groups of the cohort are visualized in Figures 5A,B.

Neurodegenerative changes occurring early in the course of AD are mainly located in the posterior parts of the brain and those observed in FTD are mainly located in frontal parts of the brain. As central structures, ventricles are adjacent to both the frontal and posterior parts of the brain, and undergo local enlargement in both AD and FTD. Displacement of ventricular surface likely integrates these atrophic changes occurring adjacent and distant to the ventricular surface and hence is a potentially sensitive marker for atrophic disease changes for use in the differential diagnosis.

## Evaluation of Predictive Value

The purpose of this study is to evaluate the predictive value of the subcortical features in the context of differentially discriminating among AD, FTD, and NC. We define the predictive value of a given feature to be the area under the curve (AUC) obtained from performing predictions on a test set unseen by the classifier in the training phase. Previous studies performed multiple pair-wise classifications in order to analyze the performance of various biomarkers such as gray matter loss, cortical thickness etc.

**Figure 4:**
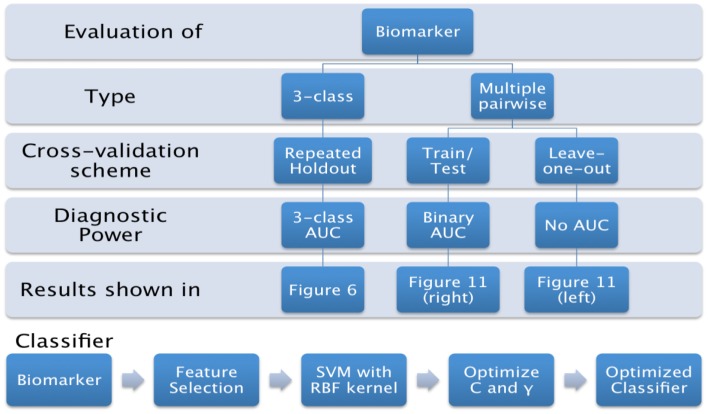
**List of experiments presented in this study, and link to the related figures**.

In this paper, we propose to evaluate the performance in a direct three-class setting, which enables a rigorous assessment of the predictive value. The three-class experiment is illustrated in Figure 6B, and the details are presented in the Section “Classifier.” For the sake of comparison, we have also evaluated the performance of the three biomarkers (volume, Laplacian invariants, and surface displacement) in the commonly used multiple pair-wise setting as well, as illustrated in Figure 6A. In each of the experiments, except for leave-one-out cross-validation (LOOCV), the class proportions were maintained in training and test sets. The detailed procedures for the two experiments are presented in the Section “Classifier.” The complete list of experiments presented this study and the link to related figures showing the results is shown in Figure 4.

**Figure 5:**
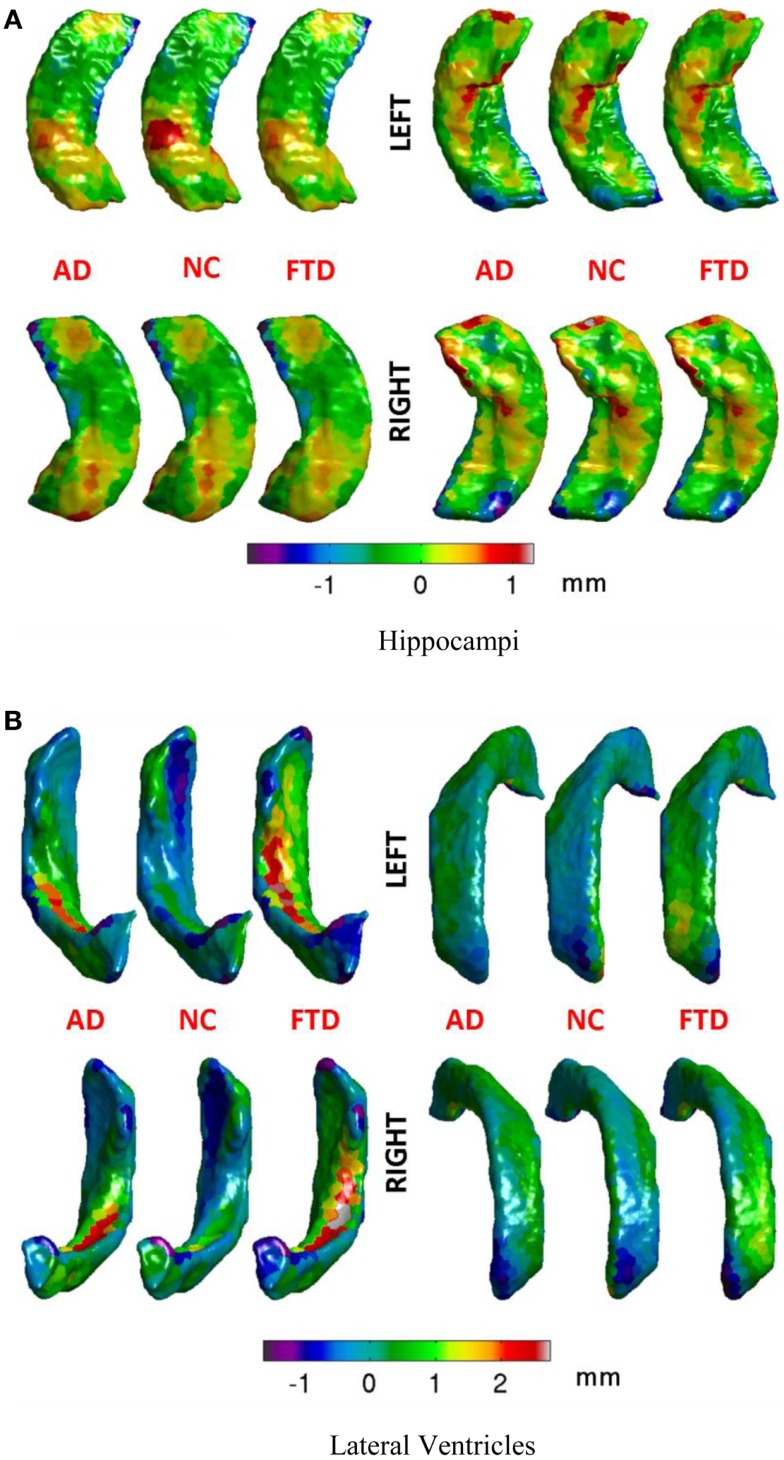
**Mean displacements in millimeters for different diagnostic groups are visualized on the average hippocampal and ventricular surfaces**. **(A)** We can see that differences in the patterns of displacements in the three groups are rather similar. We will see in the results section that this similarity is reflected in rather poor predictive value by the hippocampal displacement features. **(B)** On the ventricular surface, we can see clear differences in the patterns of displacements in the three groups. For example, in the left lateral ventricle (top left), the ventricular expansion (positive displacement in red) in FTD is relatively more anterior compared to the ventricular expansion in AD (which is relatively posterior). Also, worth noting is the lack of ventricular expansion (negative displacement in blue at anterior head) in the control group compared to both AD and FTD, which is in accordance with our understanding of the atrophy caused by these diseases. Such focal differences in the ventricular displacements make it the most predictive biomarker as will be explained later in the results section. **(A)** Hippocampi, **(B)** lateral ventricles.

**Figure 6:**
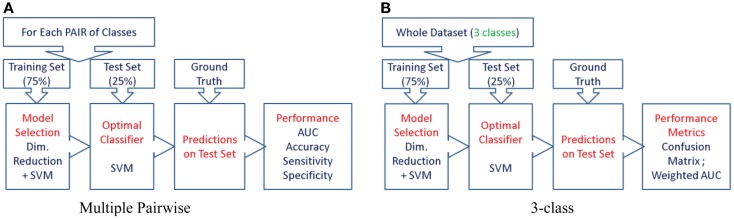
**The procedure used to evaluate the predictive value of different features is illustrated here for the two types of experiments**. We first split the cohort into the training set (75% of the cohort, or *N*-1 subjects in case of LOOCV) and use the remaining for the test set, preserving the class proportions. Although the full cohort is matched in age and gender, this might not be preserved in the training and test splits. Then, we reduce the dimensionality of the features to the top five features ranked by their information gain, which would then be passed to the SVM classifier (using a radial basis function kernel). We perform model selection for SVM on the training set only in order to obtain the most discriminative model for the particular feature. And the optimal model is used to classify the subjects from the test set (25% of the cohort, or the subject left out in case of LOOCV). This way we can assess the predictive value of a given feature. This procedure is repeated for all the features from each structure and the predictive values are evaluated. For the three-class experiment, the results are presented in Figure 7. Please note that for the multi-class experiment, we can only report accuracy and AUC. There are no metrics equivalent to sensitivity and specificity, but instead, generalized misclassification rates, as presented in Figure 8. For the multiple-pair-wise experiment, the procedure is repeated for each pair NC vs. AD, NC vs. FTD, and AD vs. FTD (and for all the features) and the results are presented in Figure 8. **(A)** Multiple-pair-wise, **(B)** three-class.

### Classifier

For both the three-class and multiple-pair-wise experiments, we have chosen to use the same classifier to evaluate the diagnostic power to enable comparison across structures as well as features. First, the dimensionality of the features is reduced to the top five features ranked by their information gain (28). These selected features are then fed to the multi-class support vector machine (SVM) classifier using a radial basis function kernel (64, 65), which uses coupling of pair-wise classifications for multi-class decisions (38). We perform model selection for SVM on the *training set alone* to optimize the penalty constant C and kernel parameter γ. The model selection is performed on the grid log_10_ *C* = -3:8 and log_10_ γ = −3:3 in order to obtain the most discriminative model for the particular biomarker. This is then applied on the test set to evaluate the performance.

As the samples size is limited and we have a large number of features for each structure, feature selection is needed to remove redundancy, reduce classifier complexity, and enhance generalization performance of the trained classifier. We have chosen to use the top five features based on an empirical relationship between the number of features used to train the classifier and the size of the sample used to avoid the curse of dimensionality (66). For *d* number of features and small probability of error *p*(*e*), the minimum sample size required is given by

$$N_{\min}\geq \frac{d}{2\;\times \;p\left(e\right)}.$$

If one would like to keep the *p*(*e*) below 5% with *d* = 5 features (after feature selection only five features are used to train the classifier), we need only 5/(2 × 0.05) = 50 subjects in total. We have 57 subjects for the training set alone and 78 subjects in the entire cohort. This ensures that sample size is sufficient and a test set can be used to estimate unbiased performance of various features.

We train the classifier treating AD and bvFTD classes to be mutually exclusive. Although AD and FTLD can co-exist, that is rare (67), and it is even rarer to find AD coexisting with bvFTD, the subtype of FTLD in this study (8).

The robustness of diagnostic performance of the proposed features to varying training and test sets are presented in the Section “Robustness to Varying Training and Testing Sets.”

### Multi-class classification

Although it is easier to perform multiple pair-wise experiments, they do not really evaluate the actual *differential diagnostic value* of the biomarkers. Hence, we propose to assess the predictive value of each biomarker directly in the three-class setting. The procedure we adopted is illustrated using a flow chart in Figure 6B.

The cohort is divided into disjoint training (for training and model selection) and test sets (for evaluation of prediction power). The training set includes 75% of the individuals from each diagnostic group and the remaining individuals in each group formed the test set. There were no specific selection criteria for training and testing. The predictive value of the each feature is then evaluated on a separate test set using the optimal model obtained from grid search (see Figure 6B). The predictive value is obtained as the weighted average of the AUC of the three receiver operating characteristics (ROC) (68, 69) generated in the three-class test, with weights for each class being proportional to its size in the test set: $w_{i}=n_{i}∕\left(\sum_{j=1}^{3}n_{j}\right),\;i=1:3$ where *n_i_* is size of the class *i* in the test set.

### Multiple pair-wise classification

We have performed two experiments in this category as described in the sections below.

#### Leave-one-out cross-validation

In this set of experiments, we have evaluated the discriminatory power of various biomarkers and also the different structures in discriminating the three pairs of diagnostic groups in a LOOCV setting. This is the most common approach found in the literature to evaluate the classification performance, *especially when the dataset consists of few samples*. The results are presented in Figure 12 (left side columns).

#### Train/test scenario

It is common knowledge in the machine learning literature that LOOCV is not the ideal way to evaluate the predictive value of a classifier (or a feature); it is *better to have a separate test set* for that purpose (40, 41). Hence, we performed this multiple pair-wise experiment to evaluate the performance of the various biomarkers and structures on an independent test set in discriminating the three pairs: NC vs. AD, NC vs. FTD, and AD vs. FTD. For each pair of classes, the cohort is divided into disjoint training set (for training and model selection) and test set (for evaluation of prediction power). The training set consists of 75% of the patients from each diagnostic group in that pair and the remaining from each group formed the test set. There were no specific selection criteria for training and testing. The procedure we adopted for this experiment is illustrated using a flow chart in Figure 6A and the results are presented in Figure 12 (right side columns).

## Results

In this section, we present the results obtained from various experiments as described in the Section “Multi-Class Classification.”

### Multi-class experiments

**Figure 7:**
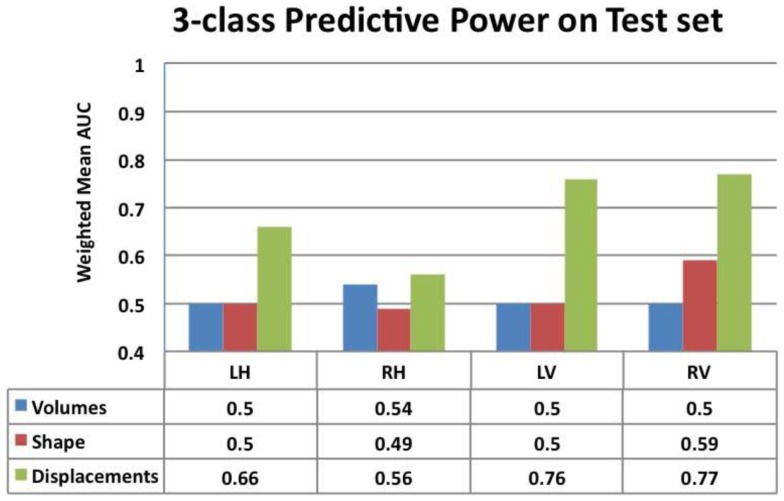
**Comparison of the predictive value – ranked by mean weighted AUC – for the volumes, Laplacian invariants, and displacements of both hippocampus and ventricles**. Here, we can see that volume feature could not discriminate groups (mean AUC of 0.5) and Laplacian invariant features performed similarly with the exception being right lateral ventricle, whose performance is slightly better than that expected by chance. The predictive value of displacements is superior to that of volumes and invariants, indicating their potential in providing differential discrimination between AD, FTD, and NC. L, left; R, right, H, hippocampus, and V, ventricle.

The procedure as illustrated in Figure 6B is repeated for each feature extracted from both hippocampus and lateral ventricle (left and right). Their performance is compared in Figure 7.

From this chart, we can see that the performance using volume feature is close to that obtained by chance (AUC ≈ 0.5), except for RH, which is slightly better than chance. The performance of the Laplacian invariant biomarker is similar to the volume feature, except for the RV. The discriminatory performance using this feature is also close to that obtained by chance, might be due to the overlapping changes caused by AD and FTD in this feature. The discrimination obtained using displacements as the feature is higher in general indicating that displacement features are more sensitive to capturing changes caused by AD and FTD. The discrimination obtained using surface displacement of ventricles is higher than that obtained from hippocampi. This indicates that the atrophy caused by AD and FTD is more distinct over the ventricular surface than over the hippocampal surface. To better understand this point, we have analyzed the group differences in ventricular surface displacements, which show significant group differences in the frontal and posterior regions of the left and right ventricle (Figure 10). This provides evidence to support the assertion that it is the differential pattern of atrophic changes in AD and FTD over the ventricular surface that is likely being captured by the surface displacement features.

Area under the curve for a feature summarizes its overall predictive value in a single number, but does not allow for a detailed evaluation of errors in classification. To further analyze the usefulness of particular features for different purposes such as screening etc., one needs to analyze the misclassification rates as well in differentiating various pairs. A confusion matrix contains aggregated information about predicted and actual classifications for a particular feature and is a square matrix of size *N*, number of diagnostic groups in the study (70). For a binary classification study, it is a 2 × 2 matrix whose terms reflect rate of false positives and false negatives and it can also be interpreted in terms of sensitivity and specificity. In a study where we have *N* = 3 or higher, we cannot define metrics like sensitivity and specificity as there are multiple classes and there is no clear way to designate which class as positive or negative. In fact, specificity and sensitivity are special names in binary classification given to generalized “correct classification rates for each class” in a multi-class problem. It is harder to define equivalent misclassification metrics when comparing multiple biomarkers. The cobweb representation (see Figures 7A,B, inspired from radar plots) is presented here as a potential solution to summarize and represent many confusion matrices into one plot. This plot has one axis corresponding to each type of misclassification that can occur in a multi-class classification study. It takes the misclassification rates from each confusion matrix (off-diagonal entries) for each biomarker and plots it on the corresponding misclassification axis.

**Figure 8:**
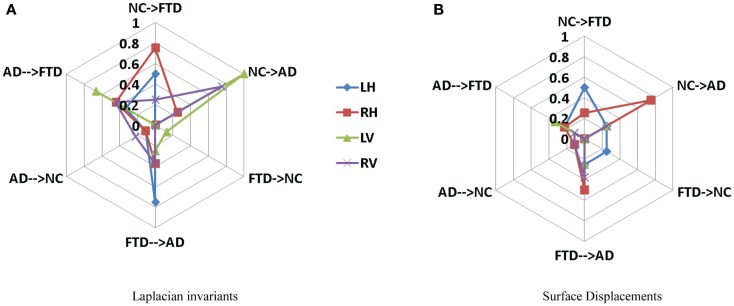
**Comparison of multi-class misclassification rates for Laplacian invariant and surface displacement features of both hippocampus and ventricles**. The cobweb plot enables an intuitive comparison of the misclassification rates for multiple features. Each axis ranges from 0 at the center (no misclassification) and extends outwards to 1 (100% misclassification). For example, the top axis “AD → FTD” refers to the percentage of subjects who were originally AD but misclassified as FTD using a particular feature. The performances of each feature (e.g., Laplacian invariants) obtained from different structures are plotted in different colors indicated by the legend. Here, L, left; R, right; H, hippocampus; and V, ventricle. **(A)** Laplacian invariants, **(B)** surface displacements.

For a three-class classification, there will be nine (3 × 3) entries in the confusion matrix for each feature, from which only six entries are plotted here on the axis corresponding to the pair being discriminated. As we can plot the misclassification for many features on the same plot, it enables an easy and intuitive comparison of the discriminatory performance of multiple features. This could prove useful in selecting features for a particular application with specific requirements. For example, in Figure 8A, the top axis “NC ≥ FTD” refers to the percentage of subjects who were originally NC but misclassified as FTD using a particular feature. Using Laplacian invariant features extracted from right hippocampus results in most misclassifications compared to the rest and using left ventricle Laplacian invariant features misclassifies none. Hence, the closer it is to the origin (center of the plot), the more accurate the associated feature in distinguishing NC and FTD. Comparing the two cobweb plots in Figures 7A,B, we can see that curves are generally closer to the origin in Figure 8B which uses surface displacement features, compared to Figure 8A which uses Laplacian invariant features suggesting that surface displacement features are comparatively more accurate.

### Multiple pair-wise experiments

The procedure as illustrated in Figure 6A is repeated for each feature extracted from both hippocampus and lateral ventricle (left and right) and the results are presented in Figure 12 (right side columns). For the sake of comparison, the same experiments are repeated using LOOCV and the results are presented in Figure 12 (left side columns).

### Robustness to varying training and testing sets

**Figure 9:**
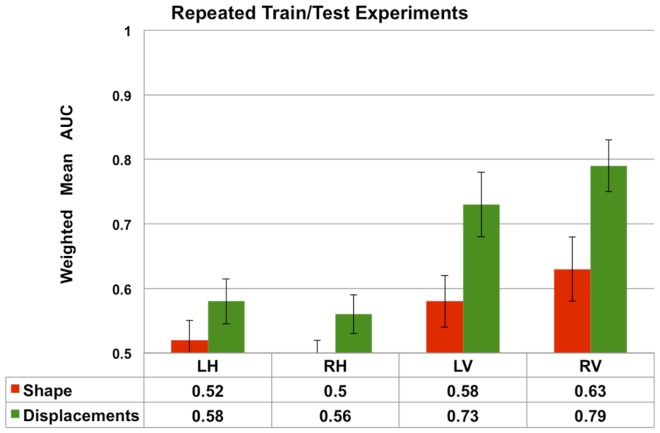
**The average performance measured by weighted mean AUC (and standard deviation shown as error bars) of the Laplacian invariant and displacement features from 10 repetitions of the train/test experiment**. In each repetition, new training (75%) and test sets are randomly generated and the experiment as described in the Section “Multi-Class Classification” is repeated. The average weighted mean AUC and the standard deviation are computed and compared in this plot. These results demonstrate the robustness of the performance of the displacement features to varying training and test sets. The performance of volume features is not shown to increase readability.

The results presented in Figures 7 and 8 are obtained from a fixed training and test sets. In order to demonstrate the robustness of the performance, we repeated the train/test experiment 10 times, each time generating new training and test sets randomly. From the 10 repetitions, the average weighted mean AUC and the standard deviation are computed and compared in Figure 9. We observe that the performance of both Laplacian invariant and displacement features, and also hippocampus and ventricles, is similar to that shown in Figure 7. The weighted mean AUC for RV improves slightly from 0.77 to 0.79. This behavior demonstrates the desirable robustness of the performance of the displacement features to varying training and test sets.

### Comparison of performance with different number of partitions

One parameter that can impact the results of this study is the number of partitions a subcortical surface is divided into. In order to study the effect, a comparison of the classification performance of the ventricular displacement features for different number of partitions (*N* = 5, 10, 20, 50, 100, 200, and 300) is presented in Figure 13.

### Comparison of different classifiers

We have chosen to employ non-linear SVM with Gaussian kernel owing to its popularity and our previous experience. For the sake of completeness, we have performed classification experiments to compare the performance of Linear SVM and Bayes Net classifier to that of non-linear SVM (with Gaussian kernel) employed in this study. This comparison is done in exactly the same way the results for non-linear SVM were obtained (feature selection, model selection, repeated hold-out CV with 10 repetitions). The results are presented in Figure 14.

## Discussion

We present the first multi-class classification study among probable AD, FTD, and NC. This study analyzes various biomarkers obtained from the hippocampus and lateral ventricles. The features compared are volumes, Laplacian invariants, and surface displacements. The study is conducted on the same cohort, separating the training and test sets, in order to compare the predictive value for these biomarkers. As the evaluation is done on a common test set, this gives valuable insight into the performance of these biomarkers. A unique feature of this study is the *design* of the three-class classification experiment and assessing the predictive value on a separate test set.

In this study, we present a novel application of Laplacian invariant features derived from the Eigen-decomposition of the Laplacian of a bounded domain (e.g., binary segmentation) adapted from application in other domains (56). We also present a template injection method to derive surface displacement features. These surface displacement features are generally large in number and present with the curse of dimensionality in the absence of a large database. We present a novel and anatomically meaningful method of reducing the dimensionality via the clustering of neighboring vertices as described in the Section “Surface Displacements via Template Injection.” This simple method, with a suitably chosen number of clusters, can be applicable for the dimensionality reduction of any boundary features of closed surface. In particular, it is well suited for feature dimensionality reduction over subcortical structures such as hippocampus and lateral ventricles.

The three-class experiment illustrated in Figure 6B resulted in the following performance for various features as shown in Figure 7. We can see that the volumetric features present with almost no discrimination (AUC ≈ 0.5), which is expected as the amount of gross atrophy caused by AD and FTD is likely similar. We can also see that the Laplacian invariants from both hippocampus and the lateral ventricles resulted in similar performance (around 0.5) suggesting that the global Laplacian invariant shape changes caused by AD and FTD are also similar. In contrary, the displacement features being a rich descriptor of local changes in atrophy outperform both the volume and Laplacian invariant features. This is probably because this biomarker contains features sensitive to spatial location on the boundary surface and allows us to separate the changes in the frontal and posterior parts of the subcortical structures (see Figure 5B), as opposed to the Laplacian invariant features that encapsulate global shape. The displacement features are also robust to varying training and test sets, as demonstrated by the results presented in Figure 9.

In addition, we have also presented a comparison of the classification performance of the displacement features w.r.t number of partitions in lateral ventricles (see Figure 13). This figure shows that the performance is reduced when the numbers of partitions are too few (*N* = 50, 10, 20), i.e., with big patches. This can possibly be due to the averaging out of the discriminating signal within the big patches. However, when the patches are sufficiently small (*N* = 100, 200, 300), the discriminatory signal is captured, leading to an improved performance. Although larger *N* (>100) led to improved performance (compared to *N* = 10, 20), the increase in performance was not proportional to increase in *N*. If we make *N* any larger, leading to patches being very small, we may end up picking up noise. This comparison shows that there is some impact of number of partitions on the results, but it is only that we need to select an optimum number of partitions to avoid washing out the discriminatory signal and at the same time to avoid picking up the noise.

In Figure 14, for the sake of completeness, we have compared the classification performance of few common classifiers that make use of ventricular displacement features. This comparison shows that the performance of the ventricular displacement features is insensitive to the choice of the classifier. The non-linear SVM is outperforming the linear counterpart, which is expected and routinely observed. Although non-linear SVM exhibits the highest performance, it is to be noted that this comparison is not exhaustive and cannot be generalized without caution.

**Figure 10:**
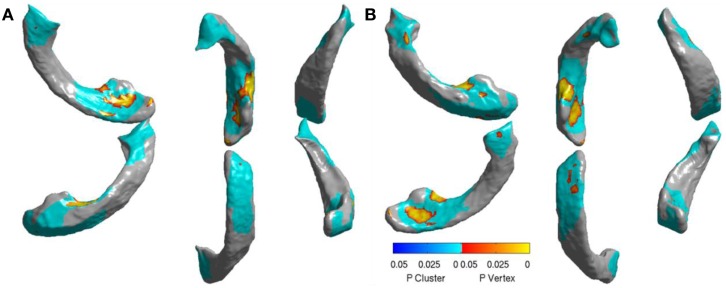
**Visualization of significant differences between AD and FTD using surface displacement features via SurfStat for the lateral ventricles – (A) the visualization above is for LV (B) and the one below is for RV**. We can clearly see that the differences are mostly localized to frontal and posterior. Please see the Section “Discussion” for further explanation.

In order to visualize the ventricular differences further, we analyzed the statistical differences between the displacements of AD and FTD using SurfStat (71). Using SurfStat, we analyzed the average group differences between AD and FTD and the set of vertices that are significantly different (*p* < 0.05 after correcting for multiple comparisons) between the two groups are presented in Figures 10A,B for left and right lateral ventricles respectively. The patches in shades of blue are set of voxels that have achieved cluster-wise significance and the vertices colored yellow to red are those that achieved vertex-wise significance. We can see that the differences are mostly frontal and posterior which supports our assertion, although our method in this study does not take advantage of this prior knowledge of disease-specific regional patterns of atrophy. This also explains why ventricular displacement features have high diagnostic value compared to that of hippocampus in this differential diagnosis study. A similar pattern of atrophy is reported in Ref. (22), where a posterior to anterior gradient of atrophy is observed in the neostriatum in AD and combined FTLD.

In order to maximize the diagnostic power, we combined the two best individual biomarkers (displacement features from LV and RV) and evaluated its three-class diagnostic power (average estimate of 3AUC from 10 repetitions). The composite biomarker exhibited 3AUC = 0.76, which did not significantly improve the performance over the individual best biomarker (RV with 3AUC = 0.765). We believe this could be due to large amount of correlation in the features from both LV and RV. We also performed another experiment to evaluate if training the SVM without feature selection, i.e., utilizing all the features (instead of only top five ranked by information gain) would improve the performance. Our results showed that not performing feature selection resulted in worse performance more often and never improved over the performance of the same biomarker with feature selection.

Previous studies in literature report results based on pair-wise classification studies (AD vs. CN, FTD vs. NC, and AD vs. FTD) as opposed to our direct three-class classification. For example, based on *pair-wise* CV experiments, 100% classification accuracy between the demented groups and their respective controls was achieved (14). This accuracy, as noted in the same report, does not represent the predictive value. Moreover, there are two sets of controls in this study in order to age-match AD group (mean age 76 years) and a much younger FTD group (mean age 67 years), whereas the cohort being studied here is age-matched well. Moreover, for differential diagnosis among dementia subtypes, a multi-class classification study would be desirable. The results reported in this multi-class classification study are obtained on a separate fixed test set, as opposed to commonly employed leave-one-out CV to reduce the variability in performance estimates (40–42, 72) and to compare the performance of different features on the same test set.

Further, as noted by the authors of Ref. (14), in the presence of three classes, a multi-class classification study would be required for differential discrimination. When a classifier is trained on two classes, e.g., AD and FTD, the classifier cannot predict if the test case is outside the classes on which the classifier was trained (for example a normal control) (38). In such a binary setting, a test case not from the AD or the FTD group would be incorrectly assigned by the classifier as belonging to the AD or the FTD group. Hence, it would be useful to develop a classifier, which can be trained on, and recognize multiple classes, concurrently, because it can also be used to classify subjects not just to those who are already diseased, but also those that are not diseased.

For the sake of comparison, we have performed multiple-pair-wise experiments using the displacement features (for both hippocampi and lateral ventricles), which proved to be the most discriminating features in the three-class setting. The performance of the displacement features in a multi-pair-wise setting is presented in Figure 12. We can see that the displacement features extracted from the lateral ventricles exhibit good discrimination among the three pairs in our study. The performance of LV (see Figure 12A) in a LOOCV setting was AUC = 0.826, 0.857, and 0.712 for the three pairs NC vs. FTD, NC vs. AD, and AD vs. FTD, respectively. It is important to note that LV demonstrated much better performance when its predictive value was evaluated using an independent test set: AUC of 0.938, 1, and 0.653 (see Figure 12B), which is more desirable. We can observe similar trends in the performance measured by AUC for RV (see Figures 12A,B).

**Figure 11:**
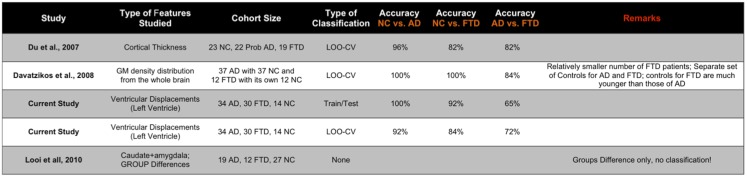
**Here, we compare the performance reported in the two other studies most relevant to the study here in terms of accuracy (ideally we would have liked to compare the AUC, but not all studies reported AUC)**. The displacement features demonstrate comparable performance even though the other studies utilize features derived from the whole brain. Note that these results are obtained from multiple-pair-wise experiments, which is neither the premise of, nor the contribution from this study. Hence, this comparison has no bearing on our three-class performance results, which are the fist-of-their kind results reported in neuroimaging literature.

**Figure 12:**
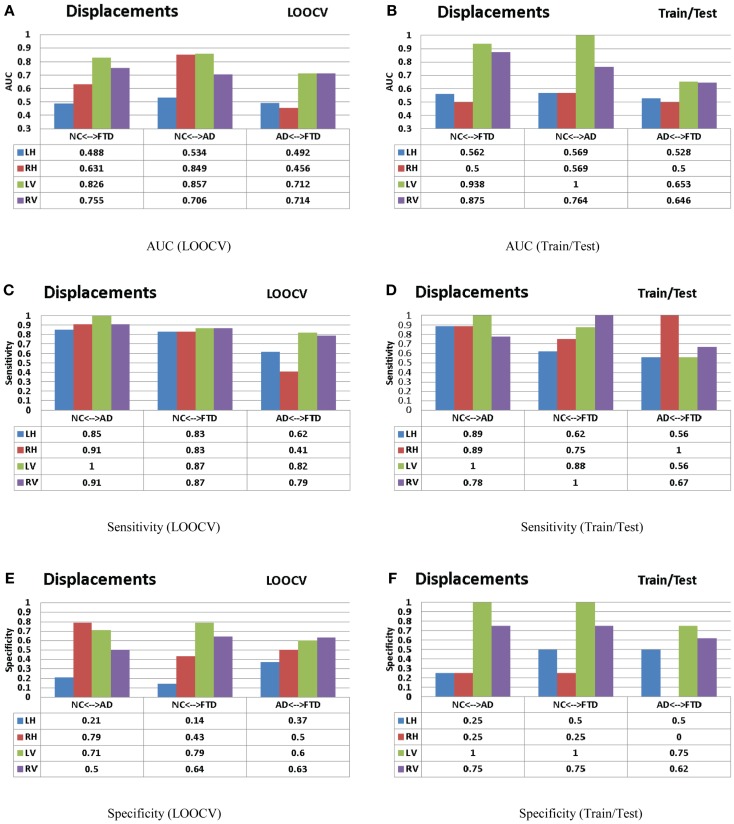
**The AUC, sensitivity, and specificity metrics obtained from the LOOCV and train/test experiments as described in the Section “Leave-One-Out Cross-Validation” (left column) and Section “Train/Test Scenario” (right column) respectively are shown here for the displacement features**. Please refer to the Section “Discussion” for description of these results. **(A)** AUC (LOOCV), **(B)** AUC (train/test), **(C)** sensitivity (LOOCV), **(D)** sensitivity (train/test), **(E)** specificity (LOOCV), and **(F)** specificity (train/test).

We have also presented the sensitivity and specificity metrics for the displacement features for the sake of completeness and comparison to previously published literature. We can see that the displacement features of both the hippocampi and lateral ventricles exhibit high sensitivity of over 0.8 and in some pairs with over 0.9 (see Figures 12C,D). We also notice from these figures that the sensitivity of these features is reduced, not surprisingly, when discriminating between AD and FTD. The results we have presented here compare to the literature (two other studies most relevant) in terms of accuracy in the manner presented in Figure 11. Ideally, we would have liked to compare the AUC (73), but not all studies reported AUC. We can see that the displacement features (derived from one subcortical structure only) demonstrate comparable performance even though the other studies utilize features derived from the whole-brain. Note that these results are obtained from multiple-pair-wise experiments, which is neither the premise of, nor the contribution from this study. Hence this comparison has no bearing on our three-class performance results, which are the fist-of-their kind results reported in neuroimaging literature.

**Figure 13:**
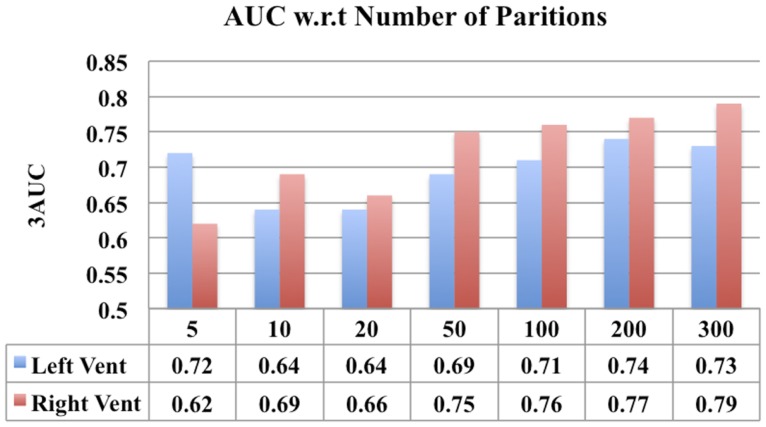
**A comparison of classification performance of ventricular displacement features (*N* = 5, 10, 20, 50, 100, 200, and 300) w.r.t number for partitions of the surface subdivision**.

**Figure 14:**
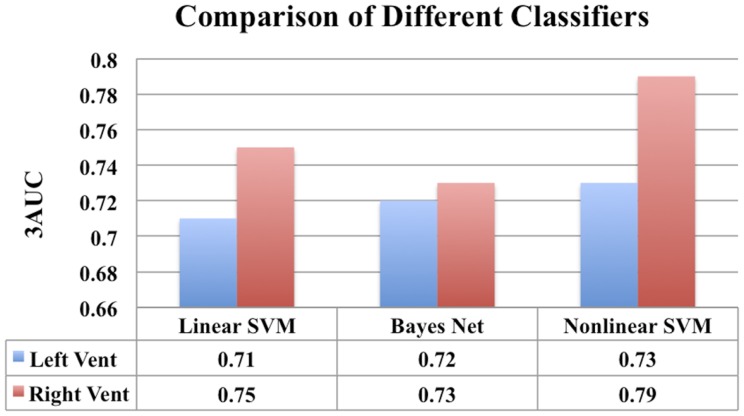
**A comparison of classification performance of different classifiers using ventricular displacement features (*N* = 300)**.

In conclusion, we present the first multi-class classification study among probable AD, FTD, and NC. The proposed novel displacement features on lateral ventricles demonstrate potential to be a reliable imaging biomarker for the three-class diagnosis task. In this model, we can easily include features from other subcortical structures such as caudate and putamen and cortical features as well. The proposed model can be easily theoretically extended to include other neurodegenerative diseases such as dementia of Lewy bodies, vascular dementia etc. with, or without, a mixed clinical presentation. But with the increasing number of diseases, the classification problem becomes increasingly harder. But this is being hindered by the practical aspects such as lack of access to uniformly collected data on the additional set of diseases, and having access to larger cohorts (with increasing number of classes) to obtain sufficient statistical power. These ideas are natural extension of presented research and will be part of our future work.

There are few limitations to our study: the diagnosis of dementia was made clinically and has not been confirmed by autopsy and hence some patients could have been misdiagnosed, although this is unlikely. Also, the severity of the disease in cohort is relatively advanced and hence the predictive performance of these biomarkers at an earlier stage of these diseases needs to be assessed in a separate study. Further, the results presented in this study are based on relatively young age of AD patients (mean age 55 years). Hence, the results may not generalize to an older population of AD patients, although they might perform better as the degeneration would be more pronounced.

### Future work

This work can be easily extended to other brain structures. Exploring the value of other subcortical structures such as caudate and amygdala would be of value to community. Combining the proposed subcortical features with complimentary features from cortical thickness and metabolic features from PET imaging, would form a natural extension of this research, that will likely improve diagnostic power in the three-class discrimination. Further studies assessing the diagnostic value of these biomarkers presented with more than three classes, e.g., including all the subtypes of FTLD as well as AD and NC would be valuable. It would also be desirable to conduct this study on a larger cohort for improved statistical power and validation.

## Conflict of Interest Statement

The authors declare that the research was conducted in the absence of any commercial or financial relationships that could be construed as a potential conflict of interest.
